# Molecular Characterization of Lys49 and Asp49 Phospholipases A_2_ from Snake Venom and Their Antiviral Activities against *Dengue virus*

**DOI:** 10.3390/toxins5101780

**Published:** 2013-10-15

**Authors:** Alzira B. Cecilio, Sergio Caldas, Raiana A. De Oliveira, Arthur S. B. Santos, Michael Richardson, Gustavo B. Naumann, Francisco S. Schneider, Valeria G. Alvarenga, Maria I. Estevão-Costa, Andre L. Fuly, Johannes A. Eble, Eladio F. Sanchez

**Affiliations:** 1 Laboratory of Biotechnology and Health, Research and Development Center, Ezequiel Dias Foundation, Belo Horizonte 30510-010, MG, Brazil; E-Mails: alzira.cecilio@funed.mg.gov.br (A.B.C.); sergio.caldas@funed.mg.gov.br (S.C.); oliveira.raiana@hotmail.com (R.A.D.O.); arthursander8@yahoo.com.br (A.S.B.S.); 2 Laboratory of Biochemistry of Proteins from Animal Venoms, Research and Development Center, Ezequiel Dias Foundation, Belo Horizonte 30510-010, MG, Brazil; E-Mails: mmikeana@yahoo.com (M.R.); gnaumann@funed.mg.gov.br (G.B.N.); schneider@yahoo.com (F.S.S.); valeria.alvarenga@funed.mg.gov.br (V.G.A.); maria.inacia@funed.mg.gov.br (M.I.E.-C.); 3 Center for Molecular Medicine, Dept. Vascular Matrix Biology, Frankfurt University Hospital, Frankfurt am Main 60590, Germany; E-Mail: eble@med.uni-frankfurt.de; 4 Department of Cellular and Molecular Biology, Federal University Fluminense, RJ 24220-008, Brazil; E-Mail: andfuly@uff.br

**Keywords:** svPLA_2_s, *Bothrops leucurus*, *Dengue virus*, antiviral effect, Real-Time PCR

## Abstract

We report the detailed molecular characterization of two PLA_2_s, Lys49 and Asp49 isolated from *Bothrops leucurus* venom, and examined their effects against *Dengue virus* (DENV). The *Bl*-PLA_2_s, named *Bl*K-PLA_2_ and *Bl*D-PLA_2_, are composed of 121 and 122 amino acids determined by automated sequencing of the native proteins and peptides produced by digestion with trypsin. They contain fourteen cysteines with p*Is* of 9.05 and 8.18 for *Bl*K- and *Bl*D-PLA_2_s, and show a high degree of sequence similarity to homologous snake venom PLA_2_s, but may display different biological effects. Molecular masses of 13,689.220 (Lys49) and 13,978.386 (Asp49) were determined by mass spectrometry. DENV causes a prevalent arboviral disease in humans, and no clinically approved antiviral therapy is currently available to treat DENV infections. The maximum non-toxic concentration of the proteins to LLC-MK2 cells determined by MTT assay was 40 µg/mL for *Bl-*PLA_2_s (pool) and 20 µg/mL for each isoform. Antiviral effects of *Bl*-PLA_2_s were assessed by quantitative Real-Time PCR. *Bl-*PLA_2_s were able to reduce DENV-1, DENV-2, and DENV-3 serotypes in LLC-MK2 cells infection. Our data provide further insight into the structural properties and their antiviral activity against DENV, opening up possibilities for biotechnological applications of these *Bl*-PLA_2_s as tools of research.

## 1. Introduction

Snake venoms have been regarded for long as excellent sources for drug discovery given their structural diversity and wide variety of biological activities [1,2,3,4]. Due to the broad range of pharmacological functions, these active components have been the subject of hundreds of scientific papers in different research fields. Snake venom PLA_2_s (svPLA_2_s) are secreted enzymes (EC 3.1.1.4) that, despite their conserved structure, these molecules have been associated with a variety of pharmacological effects [5]. They form a family of enzymes, which require Ca^2+^ and catalyze the hydrolysis of glycerophospholipids at the sn-2 position of the glycerol backbone to produce free fatty acids and lysophospholipids [6]. In addition, svPLA_2_s are small (approximately. 14 kDa), extremely stable due to the presence of seven disulphide bridges in the globular structure and have an active site histidine. These enzymes are classified into different groups based on their structural homology, disulphide pattern, catalytic specificity, and site of expression [7]. Group I of svPLA_2_s includes, the proteins mainly found in the venom of elapid and colubrid snakes (Group IA), and pancreatic svPLA_2_s (Group IB), while group II is found abundantly in the venom of viperidae snakes [5,7]. The latter group is similar to the mammalian nonpancreatic, inflammatory enzymes and are the major toxic proteins of the venom. Thus, they have a relevant role in immobilization and capture of the prey by interfering with physiological processes of victims. On the other hand, group II differs from group I in having an extended *C*-terminal tail [6,8]. 

*In vitro*, the svPLA_2_s can also modulate cell adhesion and cell proliferation and have anti-angiogenic properties [9,10,11]. Furthermore, a number of studies describe the anti-microbial properties of several svPLA_2_s against Gram negative [12] and Gram positive bacteria [13]. The readers are also referred to the recent review [5]. In addition, some svPLA_2_s or their products have been shown to interfere with viral infection, mainly by inhibiting the replication of HIV-1 and HIV-2 [14,15]. 

Over the last forty years, dengue disease has become recognized as the world’s most important mosquito-borne viral disease. Likely due to the climate change, the mosquito and the dengue virus expands into countries, which had been considered disease-free. Moreover, the virus re-emerges in countries where the disease used to be once under medical control [16]. It is the most rapidly spreading arboviral disease that is caused by four related and antigenically distinct viruses, which are named *Dengue virus* (DENV) 1–4, and belong to the *Flaviviridae* family. Dengue infection ranks in fifth position in the list of neglected tropical diseases in the Americas in terms of disability-adjusted life years (DAYLs) [17]. An estimated 2.5 billion people live in approximately 100 endemic countries and are at risk of acquiring dengue viral infection [18]. DENV is generally transmitted in a cycle involving humans and mosquito vectors, mainly *Aedes aegypti*. DENV causes a wide range of diseases in humans, from the acute febrile illness of dengue fever (DF) to life-threatening dengue haemorrhagic fever/dengue shock syndrome (DHF/DSS) [19] that can occur in hyperendemic areas with multiple circulating serotypes. Several factors such as uncontrolled urbanization and inadequate basic urban infrastructure (e.g., unreliable water supply leading householders to store water in containers close to homes) have combined to produce epidemiological conditions in developing countries in tropics and subtropics that favor viral transmission by the mosquito vector. Consequently, the number of cases of severe dengue disease continues to grow in endemic areas of Central and South America, Southeast Asia, and other subtropical regions. Children have the highest risk of developing severe disease manifestations, further underscoring the need for effective care. The total economic burden of dengue disease in the Americas was estimated to be approx. US$ 2.1 billion per year on average [20]. Since there is no specific drug therapy for DENV, the development of an effective vaccine remains a global public health priority [21].

The pit viper *B. leucurus* (white-tailed-jararaca) is a common venomous snake which inhabits restricted areas of the northeast of Brazil including the states of Ceará and Bahia to Espírito Santo in the southeast [22], where snakebite envenomations represent a relevant public health problem, *B. leucurus* is a leading cause of human accidents [23]. In a previous study, we reported the isolation and partial characterization of two svPLA_2_s from the venom of *B. leucurus*. One enzyme contains lysine at position 49 (*Bl*K-PLA_2_), and the other, an aspartic acid in this position (*Bl*D-PLA_2_). We showed that *Bl*K-PLA_2_ exhibited negligible levels of phospholipase activity as compared to that of *Bl*D-PLA_2_. In addition, *Bl*D-PLA_2_ did not affect platelet aggregation induced by ADP, collagen, and arachidonic acid, but strongly inhibited coagulation and was able to stimulate Erlich tumor growth, but not angiogenesis [24]. The absence of direct correlation between catalytic activity and biological effects has led to the hypothesis that specific effects of svPLA_2_s are due to the presence of pharmacological sites/domains on the enzyme surface [5]. The magnitude and severity of cases of the dengue disease, which acerbates with every new epidemic, spur the search for novel bioactive compounds with antiviral activities. Several studies dedicated to molecular mechanisms and structure-function relationships reported the toxicities of svPLA_2_s and their neutralization. However, little is known about the antiviral properties of these enzymes. A few reports indicate that some components of snake venoms, including svPLA_2_s, and the enzymes from bee venom have potential anti-viral activity [25]. At present, there are still no antiviral drugs being tested against dengue infection in any clinical trial. Many endeavors to develop DENV specific antiviral derivatives have been undertaken, and include the development and design of a low-cost rapid diagnostic tool, as well as the identification of safe compounds, which target multiple serotypes and are effective even after the onset of severe clinical disease [26]. In this regard, an alternative approach is to evaluate bioactive compounds such as the svPLA_2_s that could ultimately allow the advancement of lead structures for the development of a new antiviral agent against DENV. We therefore examined whether *Bl-*PLA_2_s have antiviral properties against DENV. The antiviral effects of *Bl*-PLA_2_s detected against DENV-1, DENV-2, and DENV-3 serotypes on cells in culture could be due to enzymatic activity or perhaps due to the presence of pharmacological domains on the enzyme surface, or may be due to both, that is specific protein-protein interaction and enzymatic activity. This is a topic that is under investigation by us. A model of the molecular mechanism of action of the neurotoxic svPLA_2_ ammodytoxin (AtxA) from *Vipera ammodytes ammodytes*, which includes specific protein-protein interaction, and enzymatic activity has been recently reported [27]. 

In this study, our primary objective was to determine the structural properties of *Bl*K- and *Bl*D-PLA_2_s and more closely investigate the potential of these molecules as antiviral agents against dengue virus 1–3 serotypes. Due to their binding properties, small size and high stability, the *Bl*-PLA_2_s may represent a convenient tool for proving interactions between cell surfaces. 

## 2. Results and Discussion

### 2.1. Mass Spectrometry Analysis and Sequence Determination

**Figure 1:**
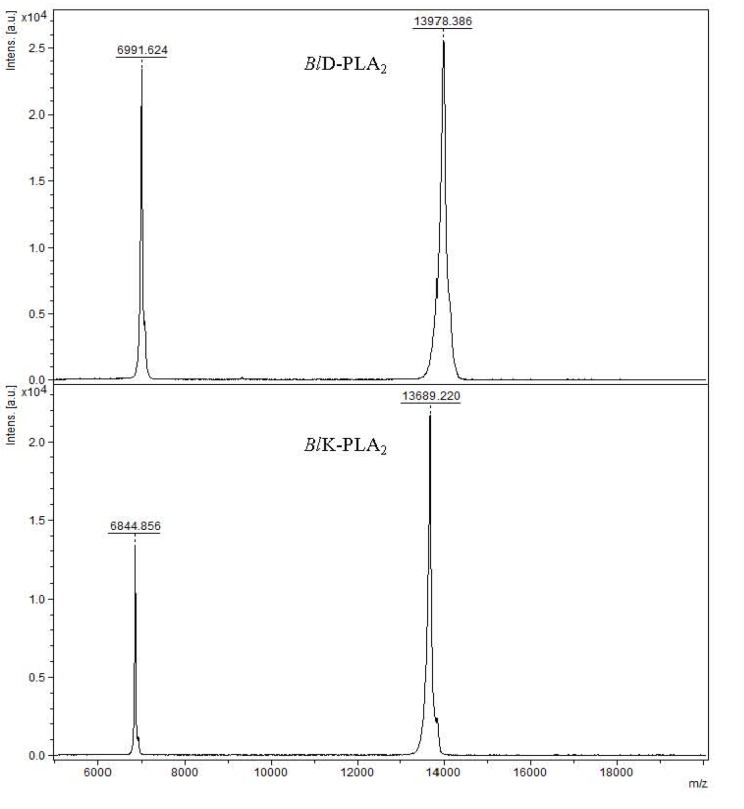
Mass spectrometry analysis of native *Bl*D-PLA_2_ and *Bl*K-PLA_2_ from *B. leucurus* venom (top and bottom panels, respectively) performed in Matrix assisted desorption/ionization-time-of flight (MALDI-TOF-MS).

**Figure 2:**
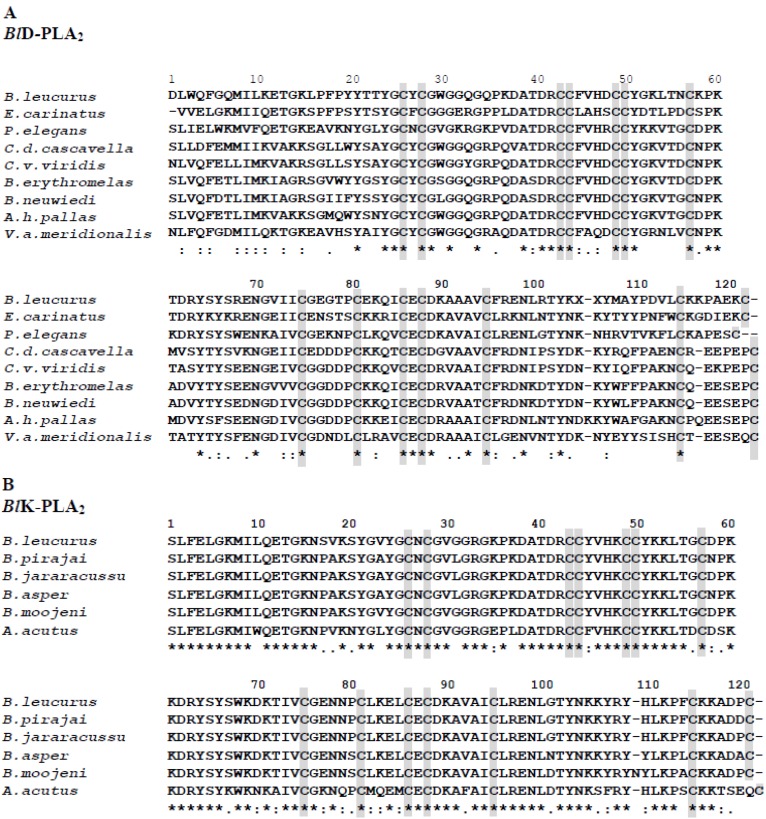
Multiple amino acid sequence alignment of *Bl*PLA_2_s with svPLA_2_s homologous. The one letter code for amino acid nomenclature is used. Proteins compared and their UniProt or GeneBank (GB) accession numbers: K49 (piratoxin II, P82287) from *Bothrops pirajai:* K49 (bthtx I, Q90249) from *B. jararacussu*: K49 (*Bl*K-PLA_2_, P86975) from *B. leucurus:* K49 (myotoxin II, P24605) from *B. asper:* K49 (myotoxin II, Q91834) from *B. moojeni:* K49 (O57385) from *Deinagkistrodon* (formerly *Agkistrodon) acutus:* R49 (Q28681) from *Protobothrops elegans:* S49 (P48650) from *Echis carinatus*; D49 (*Bl*D-PLA_2_, P86974) from *B*. *leucurus*: D49 (vipoxin complex, P04084) from *Vipera ammodytes meridionalis*: D49 (C9E7C4) from *Crotalus durissus cascavella:* D49 (Q7ZTA6) from *C.v.viridis*: D49 (Q2H228) from *B*. *erythromelas:* D49 (GB/KC544002) from *B. neuwiedi:* D49 (O42191) from *Gloydius* (formerly *Agkistrodon) halys pallas*. (*) is used for identical residues (:) for conserved ones and (.) for semi-conserved substitutions among all sequences in the alignment. Gaps were introduced to maximize the sequence homology, as indicated in part (**A**) and (**B**), respectively.

Snake venoms contain PLA_2_s isoforms exhibiting different physiological activities including antiviral properties [15,25,28]; thus, they could serve beneficial applications in medicine and as tools for biomedical research. Two *Bl-*PLA_2_s were purified to homogeneity according to the method used in previous study [24]. The MALDI-TOF mass spectrum analysis of these proteins showed - molecular masses of 13,978.386 for *Bl*D-PLA_2_ and 13,689.220 for *Bl*K-PLA_2,_ respectively (Figure 1), which fall in the typical range of svPLA_2_s. A great number of svPLA_2_s share similar structural features. The complete amino acid sequences of both enzymes have been determined (Figure 2), and deposited in the UniProt (accession numbers: P86974 for D49-PLA_2_ and P86975 for K49-PLA_2_). Sequence alignment of the isoforms was made by ClustalW program [29]. They contain 121 (*Bl*K-PLA_2_) and 122 (*Bl*D-PLA_2_) amino acid residues determined by automated Edman degradation of the reduced and pyridylethylated proteins and of peptides produced by digestion with trypsin (data not shown). Both polypeptides display a large amino acid sequence similarity between each other and to the members of Viperidae svPLA_2_s (Figure 2), including the positions of the 14 cysteine residues that may form seven disulfide bridges to stabilize the tertiary structure as proposed for homologous svPLA_2_s [5,30]. Irrespective of any enzymatic activity the overall structures of both isoforms are very similar except for the extended *C*-terminal region. The presence of two short clusters of hydrophobic/basic amino acids residues 61–71 and 105–117 (Figure 2, this work) is observed in *Bl*K49 isoform that may be the membrane-destabilizing elements as reported for Lys49 myotoxin-II from *B. asper* [31] and others. The significantly more positive surface observed for *Bl*K49- compared to the *Bl*D49-PLA_2_ isoform is in line with the higher penetrability of most basic svPLA_2_s compared to acidic and neutral enzymes [32]. 

### 2.2. Biochemical and Immunochemical Properties

**Figure 3:**
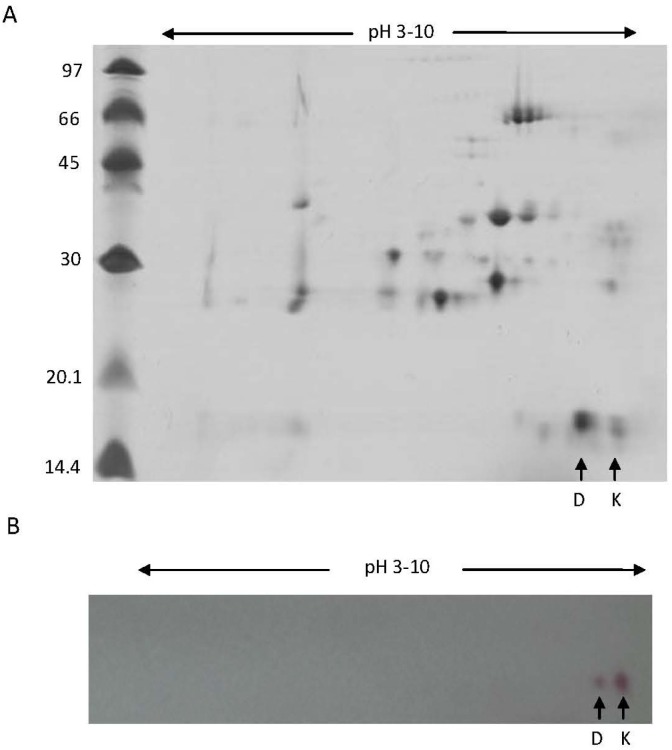
2-DE SDS-PAGE pattern of venom proteins from *B. leucurus*. (**A**) 60 µg of total proteins were isoelectrically focused (pI range 3–10) followed by separation by SDS-PAGE (15% gel) and Coomassie blue staining. Protein spots of approx. 14 kDa correspond to the position of *Bl*-PLA_2_ isoforms are indicated by arrows, (**B**) Immunoblotting of venom proteins separated by 2D-SDS-PAGE with anti-*BlK-*PLA_2_ IgG. Arrows indicates the reactivity of the antibody with the approx. 14 kDa PLA_2_s.

**Figure 4:**
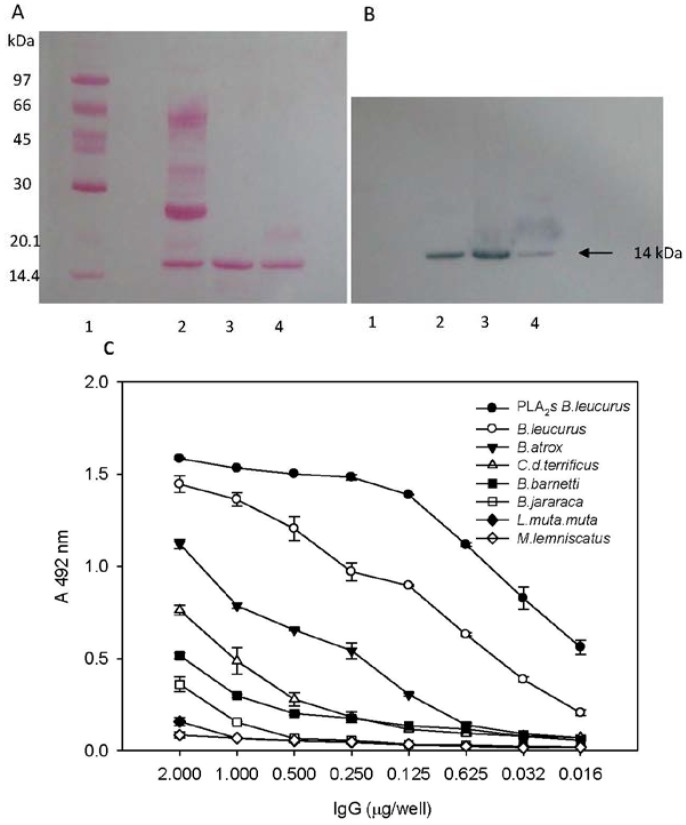
Reactivity of purified rabbit anti *Bl*K-PLA_2_ IgG against *Bl*D- and *Bl*K-PLA_2_ isoforms. (**A**) reduced SDS-PAGE (15% gel) of: 1, molecular mass markers; 2, crude venom (20 µg); 3, *Bl*K-PLA_2_ (5 µg); 4, *Bl*D-PLA_2_ (5 µg); (**B**) immunoblotting of anti *Bl*K-PLA_2_ IgG against crude venom (1), *Bl*K-PLA_2_ (2) and *Bl*D-PLA_2_ (3). (**C**) Reactivity ofanti *Bl*K-PLA_2_ IgG against several snake venoms examined by ELISA. 96-well microtitration plates were precoated with 0.5 µg/mL of *Bl-*PLA_2_s (pool), *Bothrops* species, *L. muta*, *C. d. terrificus* and *Micrurus lemniscatus*. Anti *Bl*K-PLA_2_ IgG was added at different dilutions. Binding was visualized by incubation with peroxidase-coupled anti-rabbit IgG (diluted 1:12,000) and subsequent peroxidase-catalyzed conversion of *O*-phenylenediamine (OPD). The absorbance of pre-immune serum (control) was subtracted. Data shown represent the average of two independent experiments, with error bars indicating the maximum and minimum deviation from the average.

Separated proteins of the crude venom by 2D-electrophoresis (2-DE) showed two protein spots at approx. 14 kDa (at alkaline pH), with *pI*s of 8.18 and 9.05, corresponding to *Bl*D- and *Bl*K-PLA_2_s, respectively (Figure 3A). It is known that *Bl*K49-PLA_2_s present a high isoelectric point (pI > 9) and a good stability at room temperature. Two main spots that may correspond to both isoforms were immunologically recognized by the antiserum raised against *BlK* isoform (Figure 3B). The SDS-PAGE (15% gel) analysis also showed that crude venom contain a band of 14 kDa (Figure 4A, lane 2) which correspond to the apparent molecular masses (14 kDa) of purified *Bl*K- and *Bl*D-PLA_2_s under reduced (Figure 4A, lanes 3–4) and non-reduced conditions (not shown). Furthermore, western blotting showed these bands at approx. 14 kDa of the transferred samples *Bl*K- and *Bl*D-PLA_2_s, which were immunologically recognized by the purified anti-*BlK-*PLA_2_ IgG (Figure 4B, lanes 3 and 4). In addition, a band at the same Mr (14 kDa) was immunologically detected in the crude venom (Figure 4B, lane 2). A number of svPLA_2_s interact with other proteins to form complexes or aggregates and others exist as monomers [6]. These additional molecules help svPLA_2_s to express their biological properties to the greatest potency. For instance, vipoxin, a heterodimeric post-synaptic neurotoxin found in *V. ammodytes meridionalis,* consists of two PLA_2_ subunits: a basic, highly toxic PLA_2_ and an acidic, nontoxic and enzymatically inactive PLA_2_ [33]. Furthermore, when the purified rabbit anti *BlK-*PLA_2_ IgG was tested against the *Bl-*PLA_2_s (pool) and with other snake venoms by ELISA, a strong cross-recognition was observed with *Bl-*PLA_2_s, *B. leucurus* as well as with *B. atrox*. On the other hand, *Crotalus durissus terrificus*, *Bothrops barnetti*, and *B. jararaca* gave only low cross-reactivity signals. Finally, very low cross-reaction was detected with *L.m. muta*, and there was no reaction with *Micrurus lemniscatus* (Elapidae) (Figure 4C). 

### 2.3. Phylogenetic Relationships among svPLA_2_s

**Figure 5:**
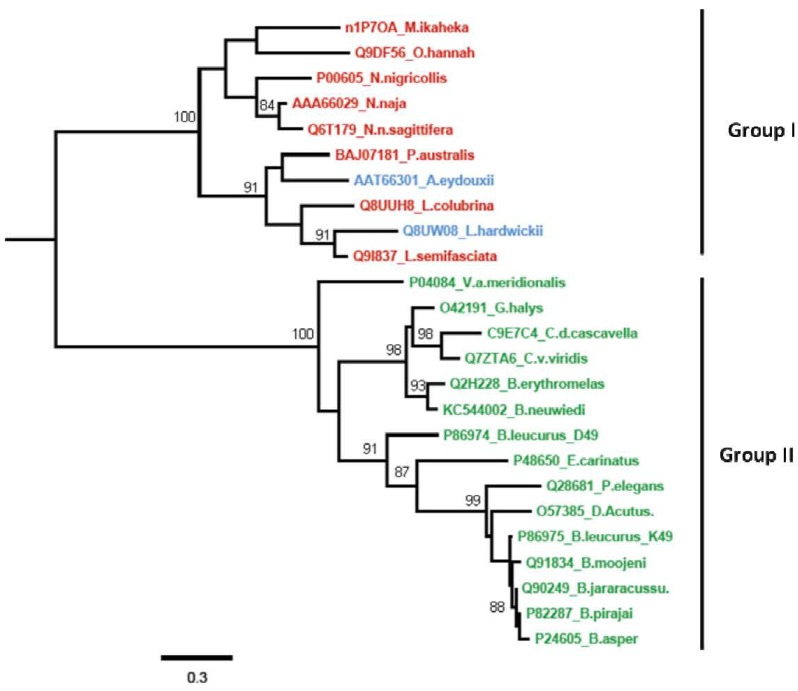
Phylogenetic tree for the multiple-sequence alignment of svPLA_2_s listed in Figure 2 (Viperidae), and some proteins from Elapidae and Hydrophiidae snakes. The length of the horizontal scale represents 30% divergence. Phylogenetic distances branch points are indicated.

A phylogenetic tree (Figure 5) was obtained by the comparison of the sequences of representative mature proteins of group II (Viperidae) listed in Figure 2, and it also includes svPLA_2_s of group I (Elapidae and Hydrophiidae) by the program phylogeny [34]. The most prominent characteristic of this tree is that the members of the different subfamilies are almost perfectly clustered and clearly separated from the other subgroup. This tree splits into two main branches, Viperidae PLA_2_s (pit vipers and true vipers) and Elapidae together with Hydrophiidae (*Lapemis hardwickii* and *Aipysurus eydouxii*) PLA_2_s, which are evidently divided. This suggests a possible evolutionary relationship between the different svPLA_2_s. Thus, the *Bl*K49 PLA_2_ (*B. leucurus*) was clustered in the same branch with homologous enzymes from *Bothrops* and *Deinagkistrodon* (formerly *Agkistrodon*) *acutus* snakes, while the *Bl*D49 isoform was clustered into the same branch with *Echis carinatus* (S49), *Protobothrops elegans* (R49), and with other Viperidae svPLA_2_s. In some members of the group II svPLA_2_s the D49 residue is replaced by serine, asparagine, or arginine and are identified as K49 [35,36]. This phylogenetic relationship appears to reflect the correlation of molecular properties and suggests that the two *Bl-*PLA_2_s represent two fundamental PLA_2_s species with different physiological activities. Phylogenetic differences of svPLA_2_s is actually the result of two independent recruitment events, elapid (group I svPLA_2_s) and viper venoms (group II svPLA_2_s) [37]. Thus, the two independently recruited types of svPLA_2_s have distinct activities, which the tissue-endogenous isoforms lacks (e.g., myotoxicity, antiplatelet effect, and neurotoxicity). In accordance with this, the toxic forms contain a positively charged hotspot on the surface that is lacking in the ancestral form from the pancreas [38]. 

### 2.4. Antiviral Assays

**Figure 6:**
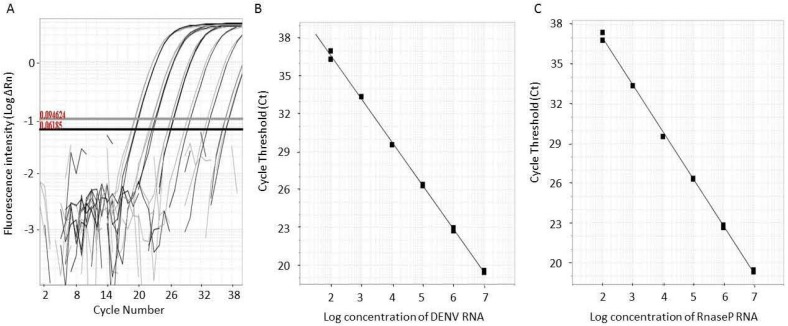
DENV and RnaseP standard curves generated from transcribed RNAs. (**A**) Curves were generated from seven serial dilutions of transcribed DENV and RnaseP RNAs from 10^7^ copy number/µL. The black lines refer to DENV RNA and the gray lines to RnaseP RNA (exogenous control). (**B**,**C**) Standard curves were generated from the linear region of each amplification curve. Efficiency of amplification for each primer set was determined using the equation: Efficiency (*E*) = 10^(−1/sl^°^pe),^ being *E*_DENV_ = 91.34%, *R*_DENV_ = 0.999, *E*_RnaseP_ = 95%, *R*_RnaseP_ = 0.999.

In this study we evaluated the antiviral activity of two isolated svPLA_2_s. To this end a one-step qRT-PCR was employed using a TaqMan technology with a probe for DENV detection and a probe for exogenous control (RNAseP) detection in a single multiplex assay. The use of qRT-PCR was performed in this study to avoid the risk of cross contamination and to increase the speed of the assay [39]. The addition of an amplification control (RNAseP) was particularly useful to monitor possible false negative results (due to RNA degradation) and to correct variations in the amounts of initial samples (due to different RNA recovery and samples loading), thus enabling PCR data normalization [40,41]. The PCR was calibrated *in vitro* RNAs transcripts of viral and exogenous control (RnaseP). The DENV and RnaseP standard curves were generated from five serial dilutions of transcribed RNAs from 10^7^ copies/µL. The detection limit of the assay was 10^2^ copies/reaction. Figure 6A shows amplification curves of DENV and RnaseP in five log dilutions. Figure 6B,C show the standard curves generated from the linear region of DENV and RnaseP amplification curves. The efficiency (*E*) and Pearson Correlation coefficient (*R*) value for both curves were: *E*_DENV_ = 91.34%, *R*_DENV_ = 0.999, *E*_RnaseP_ = 95%, *R*_RnaseP_ = 0.999. These high efficiencies (>90%) and *r* value (0.999) were important features of the quantitative Real-Time PCR strategy developed for our *in vitro* assays. High and similar PCR efficiencies of the target of interest and the normalization of reaction are important prerequisites for an accurate quantification, but not always considered in different studies using real-time PCR available in the scientific literature. 

**Figure 7:**
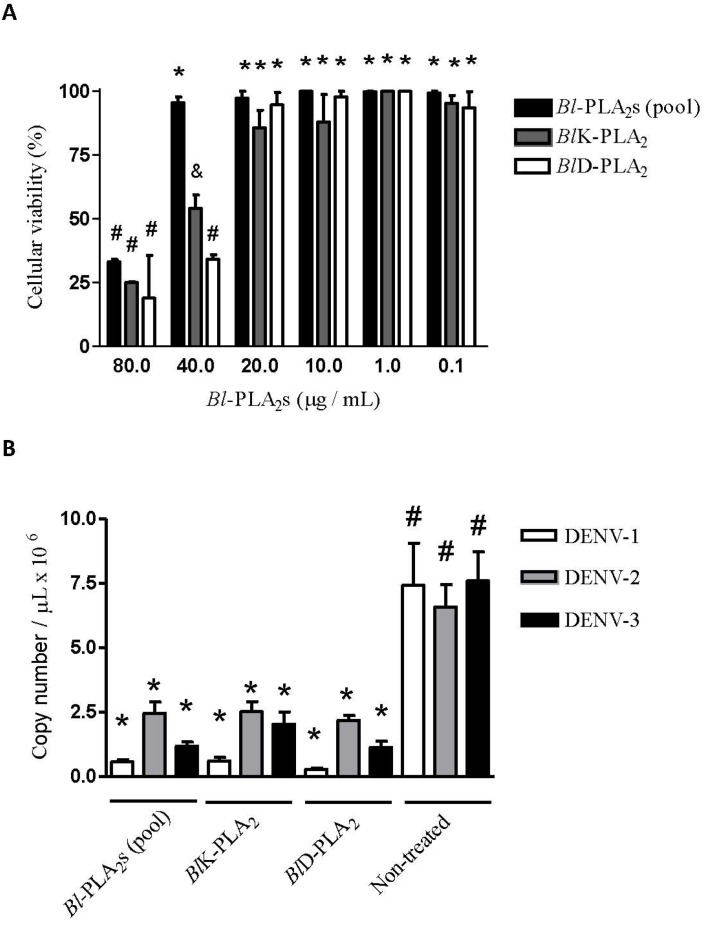
(**A**) Cellular viability after the incubation of LLC-MK2 cells with different concentrations of *Bl*-PLA_2_s (Pool) and their isoforms *Bl*K- and *Bl*D-PLA_2_ for 48 h. The higher concentrations without significant toxicity (*p* < 0.05) were considered as the maximum non-toxic concentration (MNTC). Data represent the mean of three independent experiments performed in triplicate. Means with different symbols denote significant differences (*p* < 0.05). (**B**) Antiviral activity assessed by quantitative Real-Time PCR. Data represent the mean of two independent experiments performed in triplicate.

To carry out the *in vitro* antiviral assays, we first determined the concentration at which the *Bl-*PLA_2_s and their isoforms could be used (to evaluate their antiviral effects) without causing damage to the cell culture. The maximum non-toxic concentration (MNTC) determined by cell viability was 40 µg/mL for *Bl-*PLA_2_s (pool, partially purified fraction containing both PLA_2_s isoforms) and 20 µg/mL for each enzyme *Bl*K- and *Bl*D-PLA_2_ (Figure 7A). The lack of cytotoxicity of the pool fraction at 40 µg/mL may indicate the presence of any compounds that interfere with the activity of *Bl*-PLA_2_s.Then, antiviral assays were conducted to evaluate the ability of *Bl-*PLA_2_s (pool) and the enzymes, without catalytic (*Bl*K-) and with catalytic activity (*Bl*D-PLA_2_) to inhibit the multiplication of DENV in LLC-MK2 cells compared to non-treated cells. The viral load was determined by qRT-PCR quantification of DENV RNA copies. Cells treated with *Bl-*PLA_2_s (pool), as well as cells treated with each isoform (*Bl*K- and *Bl*D-PLA_2_)_,_ showed a significant reduction (*p* < 0.05) in the number of DENV-1, DENV-2, and DENV-3 RNA copies when compared to non-treated infected cells. Similar results were obtained with all DENV used and no significant differences were observed (*p* < 0.05) in the antiviral assays of *Bl-*PLA_2_s (pool), *Bl*K- and *Bl*D-PLA_2_ (Figure 7B). 

Brazil is the country with highest number of cases and highest cost, but the economic impact of dengue is substantial in many American countries, with the cost per capita greater than US$ 2.1 billion per year in four of the six American subregions (Andean region, Brazil, the Caribbean, and Central America and Mexico) [18,20]. Interestingly, treatment of the cell cultures after viral adsorption did not result in significant inhibitory effect on the viral replication (data not shown). This observation points to an effect of phospholipases in the cell membrane level, since svPLA_2_-application after viral penetration into the cell did not reduce load in infected cells as compared to untreated cells. An antiviral effect showed by svPLA_2_ from *C. d. terrificus* against DENV-2 and yellow fever virus (YFV) was recently reported [25].

## 3. Materials and Methods

### 3.1. Purification of BlK-PLA_2_ and BlD-PLA_2_

Two *B.leucurus* snakes were captured in the north of the Minas Gerais State and kept at the serpentarium of the Ezequiel Dias Foundation (FUNED, Belo Horizonte, Brazil). Venoms from these captive specimens were collected manually by milking. The pooled samples of the freeze-dried venoms were stored at 4 °C. *Bl*-PLA_2_s enzymes were purified as described in a previous work [24]. The homogeneity of purified svPLA_2_s was determined by using MALDI-TOF mass spectrometry, SDS-PAGE under reducing and non-reducing conditions and by *N*-terminal sequence determination. All protein concentrations throughout this study were determined using a BCA assay kit (Pierce Chemical Company, Rockford, IL, USA). Anti-*BlK*-PLA_2_ antiserum was raised in a rabbit (New Zealand 2.3 kg) using as antigen the isoform K49 as described [42]. The IgG fraction of immune rabbit serum was purified by affinity chromatography on protein A-Sepharose. The experiments reported here were performed in accordance with the guidelines established by the Brazilian College for Animal Experimentation and approved by FUNED Animal Ethics Committee. 

### 3.2. Protein Characterization

The molecular masses (*M*r) of purified *Bl*K- and *Bl*D-PLA_2_s were determined by SDS-PAGE (15% gel) and by MALDI TOF mass spectrometry. The *N*-terminal and the complete amino acid sequence of each protein was determined by automated Edman degradation using a Shimadzu PPSQ-21A protein sequencer according to the manufacturer’s instructions. 

### 3.3. MALDI-TOF Mass Spectrometry

Protein masses were determined by Matrix assisted laser desorption/ionization-time-of flight (MALDI-TOF) mass spectrometry. Spectra were recorded and analyzed using a Bruker Autoflex III Smartbeam instrument in the linear positive mode controlled by the proprietary COMPASS™ 1.2 software package. The Nd-YAG-laser power (355 nm) was manually adjusted for optimal signal appearance. A freeze-dried salt- and detergent-free sample was dissolved in few microliters of 30% ACN in 0.1% TFA. 0.5 µL was spotted on a ground steel target plate, mixed with 0.5 µL matrix solution (10 mg/mL sinapinic acid in 50% ACN, 0.1% TFA) and left to dry at room temperature. Commercially available standard protein mixtures were spotted on the same target for calibration purposes prior to sample analyses. 

### 3.4. 2D-Electrophoresis (2-DE) and Image Analysis

The proteins (60 µg) of *B. leucurus* venom were separated by 2-DE. Prior to running the first dimension, the IPG strips were placed in the rehydration tray and the proteins were dissolved in the De Streak Rehydrate solution (GE Health Care, Uppsala, Sweden), 0.5% IPG buffer pH 3–10 (GE Health Care). First dimension IEF was carried out in an Ettan IPGphor 3 (GE Health Care) as described by the manufacturer. Immobiline strips 7 cm, pH 3–10 linear (GE Health Care) were employed for the first dimension separation at 20 °C using a four-phase electrophoresis program: 300 V to reach 200 Vh; 1000 V to reach 300 Vh; 5000 V to reach 4000 Vh; and 5000 V to reach 1250 Vh (total accumulated 5800 Vh with 50 µA/strip). Prior to running the second dimension the proteins in the strip were reduced and alkylated by sequential incubation in the following solutions: 75 mM Tris pH 8.8, 6 M urea, 3% glycerol, 2% SDS, 0.002% bromophenol blue (equilibration buffer-EB), 10 mg/mL DTT for 20 min; and then a solution of 25 mg/mL iodoacetamide in EB for 20 min. In addition, SDS-PAGE was done in a mini gel 7.5 cm 15% polyacrylamide gel. Proteins were visualized using Coomassie blue staining. Direct scanning and image analysis was performed using an Image Master 2D Platinum 7 (GE Health Care). 

### 3.5. Protein Sequencing

The thiol groups of purified proteins (1.5 mg each) were S-reduced and alkylated with vinyl pyridine as described [43]. The material was dissolved in 1 mL of 0.1 M Tris-HCl (pH 8.6), 6 M guanidine-HCl. After addition of 30 µl β-mercaptoethanol the samples were incubated first at 50 °C for 4 h under nitrogen, then with 40 µL of 4-vinyl pyridine in the dark at 37 °C for 2 h and subsequently desalted on a Vydac C4 column with a gradient of 0%–60% acetonitrile in 0.1% TFA. The *S*-pyridylethylated (PE) proteins were digested with trypsin (2% *w*/*w*, enzyme:substrate in 1 mL of 0.1 M ammonium bicarbonate, pH 7.9) for 3.5 h at 37 °C. The cleavage products were separated on a Vydac C18 small pore column (4.6 × 250 mm) in a linear gradient of 0%–50% acetonitrile in 0.1% aqueous TFA and sequenced using a Shimadzu PPSQ 21A protein sequencer. The primary structures of *Bl*K- and *Bl*D-PLA_2_s were compared with the sequences of other related proteins in the SWISS-PROT/TREMBL data bases using the FASTA and BLAST programs.

### 3.6. Immunoblot and Enzyme-Linked Immunoabsorbent Assay (ELISA)

For analysis of immunological reactivity of several different snake venoms against the anti-*BlK*-PLA_2_ IgG, western immunoblotting and ELISA were used. The *Bl*K- and *Bl*D-PLA_2_s isoforms (4 µg each) was subjected to SDS-PAGE (15% gel) under reducing conditions, then eletrophoretically transferred onto a nitrocellulose membrane according to the manufacturer’s (Bio-Rad, Richmond, CA, USA) instructions. One gel of 2-DE containing separated proteins from *B. leucurus* venom was also used for immunoblot assay under similar experimental conditions. ELISA plate was coated with 100 µL of 0.5 µg/well of each antigen (*Bl*-PLA_2_s or crude venoms) in 0.05 M carbonate buffer, pH 9.6. After washing with 0.05% Tween-saline, a blocking solution (2% casein in phosphate buffered saline-PBS) was added (1 h at room temperature). After two washes with the same solution, anti-*BlK*-PLA_2_ antibody previously diluted in PBS containing 0.25% casein and 0.05% Tween 20 (0.015 to 2 µg/well) was added and incubated for 1 h at 37 °C. After six washes, peroxidase-coupled anti-rabbit IgG (Sigma, St. Louis, MO, USA, diluted 1:12,000) was added and incubated for 1 h at room temperature. The wells were washed and 100 µL of peroxidase substrate OPD (0.33 mg/mL in citrate buffer, pH 5.2, in the presence of 0.012% H_2_O_2_) was added and the color reaction developed for 1 h in the dark. Absorbance was read with a micro-plate reader at 492 nm.

### 3.7. Cells and Viruses

*Aedes albopictus* cells (C6/36 Line, mosquito cells) were maintained in Leibovitz’s medium (L-15, Gibco/Invitrogen) supplemented with 10% fetal bovine serum (FBS) (Gibco/Invitrogen), 100 U/mL penicillin G, and 100 µg/mL streptomycin (Gibco/Invitrogen) at 28 °C in a B.O.D. incubator. Rhesus Monkey Kidney Epithelial Cells (LLC-MK2 Line) were maintained with Dulbecco’s modified Eagle’s medium (DMEM) supplemented with 5% FBS, 100 U/mL penicillin G and 100 µg/mL streptomycin at 37 °C in a humidified 5% CO_2_ atmosphere. The viruses DENV-1 (Brasil/98), DENV-2 (SpH 125367) and DENV-3 (RibH 1) were propagated in C6/36 cells and titrated using Dulbecco plaque technique [44] in LLC-MK2 cells. Aliquots were stored at −80 °C until required.

### 3.8. Determination of the Non-Cytotoxic Concentrations

The non-cytotoxic concentrations of the compounds *Bl-*PLA_2_s (Pool, partially purified fraction containing both PLA_2_s) and their isoforms *Bl*K- and *Bl*D-PLA_2_s were determined by assessment of cell viability using the MTT [3-(4,5-dimethylthiazol-2-yl)-2,5-diphenyl tetrazolium bromide] assay (Sigma). Confluent LLC-MK2 cells monolayers (4 × 10^4^ cells/well) in 96-well microplates were exposed to different concentrations (0.1, 1.0, 10.0, 20.0, 40.0, and 80.0 µg/mL) of *Bl-*PLA_2_s (Pool), and the purified enzymes for 48 h in a CO_2_ incubator. After the incubation period, cells were observed using an inverted optical microscope (Nikon, Tokyo, Japan) and washed twice with PBS buffer (136.9 mM NaCl, 2.68 mM KCl, 8.1 mM Na_2_HPO_4_, 1.47 mM KH_2_PO_4_, pH 7.4). Then, 100 µL of MTT solution (0.5 mg/mL in DMEM) was added to each well and the plate was maintained in a CO_2_ incubator. After 3 h of incubation at 37 °C the plates were centrifuged at 400 × *g* for 10 min. The supernatant was removed and 50 µL of dimethyl sulfoxide (DMSO) was added to each well to solubilize the formazan crystals. The absorbance was measured at 540 nm using an automated Microplate Reader, Pharmacia Biotech Ultrospec 1000. The cellular viability was calculated by comparison with untreated cells, which was set to 100% viability. Each data point was the mean value of triplicates from three independent experiments. The highest concentration of each compound without any significant toxicity (*p* < 0.05) was considered as the maximum non-toxic concentration (MNTC).

### 3.9. Antiviral Assay

Antiviral assays were conducted to evaluate whether any *Bl-*PLA_2_s pool and/or isolated proteins could inhibit the multiplication of DENV in LLC-MK2 cells. *Bl-*PLA_2_s was added at their maximum non-toxic concentrations (MNTCs) to confluent LLC-MK2 cells monolayers (1.9 × 10^5^ cells/well) in 24-well plates. Afterwards, DENV-1 was added at a multiplicity of infection of 0.05 to treated and untreated cells, and the plates were incubated for 48 h in a 5% CO_2_ humidified incubator. After this period, the cell supernatants were collected and submitted to RNA extraction for viral load quantification by quantitative Real-Time PCR (qRT-PCR). Two independent experiments were performed in triplicate. The antiviral assay was repeated using two different DENV serotypes (DENV-2 and DENV-3).

### 3.10. RNA Extraction

RNA was extracted from the supernatants (140 μL) of antiviral assays using QIAamp^®^ Viral RNA Mini Kit (Qiagen, Hilden, Germany), following the manufacturer’s protocol. An exogenous control (10 μL of cloned RNase P, corresponding to 1 × 10^5^ RNA copies) was added to each sample prior to RNA extraction. Finally, the RNA was eluted in 60 μL of diethyl pyrocarbonate (DEPC) treated water (Sigma) and maintained at −80 °C.

### 3.11. Generation of RNA Standard

Using pGEM-T Easy vector (Promega, Madison, WI, USA), two plasmids were constructed by cloning the 67 bp amplicon of 3'UTR region from DENV-1, consensus to the four serotypes of DENV [45], and 64 bp amplicon from Human RNAseP [46]. The amplicons were generated by SuperScript^®^ III Platinum One-Step qRT-PCR System (Invitrogen, Carlsbad, CA, USA) using the primers shown in Table 1, according to the manufacture’s instructions. The presence and orientation of the insert DNA were confirmed by sequencing. The plasmids were linearized by digestion with Nde I and the target sequences were amplified using MEGAscript^®^ High Yield Transcription Kit (Ambion, Austin, TX, USA). The transcribed RNAs were treated with DNAse (Invitrogen) and purified using the MEGAclear™Kit (Ambion). The RNAs were quantified by spectrophotometry (Genequant, Amersham Pharmacia Biotec, Cambridge, England). The copy numbers of the RNAs were calculated based on the concentrations and their molecular weights and 10-fold serial dilutions of these RNAs, ranging from 10^7^ to 10^3^ copies/µL were used as standards in all qRT-PCRs. The detection limit of the assay was verified from successive dilutions of the standards until detection was impossible or inaccurate. 

**Table 1:** Probes and primer sequences for Dengue virus, DENV (3'UTR) and exogenous control (RnaseP) used in quantitative Real-Time PCR assay.

Target	Sequence (5'-3')	Nucleotide position
3'UTR-F	GARAGACCAGAGATCCTGCTGTCT	10,647–10,670
3'UTR-R	ACCATTCCATTTTCTGGCGTT	10,714–10,694
3'UTR-Probe	VIC-AGCATCATTCCAGGCAC-MGB-NFQ	10,675–10,691
RnaseP-F	AGATTTGGACCTGCGAGCG	50–68
RnaseP-R	GAGCGGCTGTCTCCACAAGT	114–95
RnaseP-Probe	FAM-TTCTGACCTGAAGGCTCTGCGCG-BHQ1	71–91

### 3.12. Quantitative Real-Time PCR

Quantitative Real-Time PCR was performed using a Real-Time PCR System StepOnePlus (Applied Biosystems, Foster City, CA, USA) and SuperScript™ III Platinum^®^ One-Step qRT-PCR System (Invitrogen). Amplifications were carried out in reaction mixtures containing 5 μL of transcribed or viral RNA, 0.5 μL of SuperScript™/Platinum^®^ Taq Mix, 2x reaction mix buffer, 0.4 μM of primers to DENV, 0.2 μM of primers to RnaseP, 0.2 μM of TaqMan probe to DENV, 0.1 μM of TaqMan probe to RnaseP (Applied Biosystems) (see Table 1), 0.25 μL of ROX Reference Dye, 0.5 μL of RNaseOUT™ (Invitrogen), and DEPC-treated water to 25 μL final volume. The cycling program consisted of an RT step at 50 °C for 30 min, initial denaturation at 95 °C for 2 min, followed by 40 cycles at 95 °C for 15 s and 60 °C for 1 min. Each reaction set was checked for contamination using negative control (all reagents included and water instead of DNA). In addition to this negative control, cells not infected were included in the assay. 

### 3.13. Statistical Analysis

Data were expressed as means ± standard error (SE). Statistical differences were determined by the nonparametric Tukey’s Multiple Comparison Test. Pearson’s correlation (*R*) was used to evaluate the relationship between Cycle threshold (*C*t) and quantity of RNA on standard curves of qRT-PCR. Values of *p* < 0.05 were considered significant.

## 4. Conclusions

We have described the main structural properties of two basic svPLA_2_s, *Bl*K- without and *Bl*D-PLA_2_s with hydrolytic activity from *B. leucurus* venom. The promising results observed in our antiviral assays, with decreased amounts of viral RNA quantified in cells treated with *Bl-*PLA_2_s, opening up possibilities for biotechnological applications of these molecules as research tools. Furthermore, the data suggest that, in addition to catalytic activity, the physiological role of *Bl*PLA_2_s can be mediated by ligation to specific receptors on the cell membrane, which is in accord with the higher penetrability of most basic svPLA_2_s compared to neutral and acidic enzymes [36] and others. Thus, consistent with these findings, *Bl*-PLA_2_s display cytotoxic properties against DENV *in vitro*, suggesting that they are useful tools against DENV or as a prototype to develop anti-dengue drug. 
